# Real-world study of first-line immunotherapy combined with chemoradiotherapy in esophageal squamous cell carcinoma

**DOI:** 10.1038/s41598-025-12250-w

**Published:** 2025-08-04

**Authors:** Xiaohan Zhao, Chengang Weng, Wuhan Yang, Hongmei Gao, Hesong Wang, Wenzhao Deng, Shuchai Zhu, Wenbin Shen

**Affiliations:** 1 Department of Radiation Oncology, The Fourth Hospital of Hebei Medical University Cancer Institute, No. 12 Jiankan Road, Chang’an District, Shijiazhuang, 050011 China; 2 Department of Thoracic Surgery Department, The Fourth Hospital of Hebei Medical University Cancer Institute, Shijiazhuang, China; 3 Department of Hepatological Surgery Department, The Fourth Hospital of Hebei Medical University Cancer Institute, Shijiazhuang, China; 4 Department of Radiation, Shijiazhuang People’s Hospital, Shijiazhuang, China

**Keywords:** Immunotherapy, Radiotherapy, Chemotherapy, Esophageal squamous cell carcinoma, Prognosis, Treatment-related adverse events, Cancer immunotherapy, Cancer microenvironment, Cancer therapy

## Abstract

This study aimed to assess the effectiveness, safety, and recurrence patterns of first-line immunotherapy combined with chemoradiotherapy in esophageal squamous cell carcinoma (ESCC) patients. A retrospective analysis of 79 eligible ESCC patients was conducted. Primary outcomes included overall survival (OS) and progression-free survival (PFS), with secondary outcomes being objective response rate (ORR), disease control rate (DOR), treatment-related adverse events (trAEs), and treatment failure patterns. The median follow-up was 29.4 months, with median OS unreached and median PFS of 14.6 months (95% CI 10.7–18.5). ORR was 82.3%, and DOR was 96.2%. Factors affecting OS were clinical stage, immunotherapy cycles, immunotherapy and chemoradiotherapy sequence, radiation coverage, mid-treatment lymphocyte count, and short-term efficacy (HR = 2.254, 0.374, 2.653, 2.957, 2.309, 2.789; *P* = 0.030, 0.019, 0.009, 0.004, 0.001, 0.014). Factors impacting PFS were clinical stage, immunotherapy and chemoradiotherapy, and post-treatment lymphocyte count (HR = 2.135, 2.048, 1.911; *P* = 0.007, 0.010, 0.001). Among the cohort, 46.8% experienced treatment failure, with 33 receiving second-line treatment, resulting in a median OS of 14.17 months (95% CI 7.303–21.037) and 1- and 2-year OS rates of 56.7% and 24.3%. Notably, 36.7% experienced grade ≥ 2 trAEs, bone marrow suppression most commonly happened, and 5.1% developed esophageal fistulas. Immunotherapy combined with chemoradiotherapy demonstrates strong anti-tumor activity and tolerability in ESCC patients, The radiation for all leisions, sequential immunotherapy combined with chemoradiotherapy, and higher levels of lymph node cell counts are associated with a better prognosis. Large-scale randomized controlled trials are needed for further validation.

## Introduction

Esophageal squamous cell carcinoma (ESCC) is one of the major health concerns affecting the Chinese population^1^. Unfortunately, a substantial proportion of patients are diagnosed at an advanced clinical stage, thereby missing the opportunity for curative surgical treatment. Chemoradiotherapy (CRT) is the standard treatment for inoperable ESCC^2^, but local recurrence and distant metastasis after treatment contribute to poor prognosis, occurring in over 50% of cases^3^. Therefore, there is an urgent need for novel anticancer agents to improve the prognosis of locally advanced or metastatic esophageal cancer patients who are not eligible for surgical resection.

Recent years, immune checkpoint inhibitors (ICIs) have shown significant improvements in the prognosis of ESCC patients^4–9^^.^ However, most evidence is derived from clinical trials, especially data on the combination of chemotherapy and immunotherapy in ESCC, which is still in the experimental stage, lacking data from real-world clinical application. Additionally, the absolute lymphocyte count (ALC) has been identified as a crucial prognostic factor for malignant tumor patients, with clinical validation^10,11^. However, its predictive value for the prognosis of ESCC patients receiving ICIs in combination with CRT has been rarely reported. In this retrospective real-world study, we not only analyze the efficacy, adverse events, and recurrence patterns of ICIs in combination with CRT for unresectable locally advanced or metastatic ESCC patients but also investigate the impact of ALC levels at different time points and radiation field on patient prognosis.

## Material and methods

### Ethical approval

Ethical approval was obtained from the Ethics Committee of the Fourth Hospital of Hebei Medical University (Ethical approval number: 2023KS004) and conducted in accordance with the practice of the Declaration of Helsinki and relevant guidelines, regulations and policies required by the journal. Informed consent was waived by the Ethics Committee of the Fourth Hospital of Hebei Medical University due to the retrospective nature of the study.

### Inclusion and exclusion criteria: inclusion criteria

(1) Patients diagnosed with ESCC by pathological examination; (2) Staged as II to IV according to the 8th edition of the American Joint Committee on Cancer (AJCC) TNM staging, (3) Without prior antitumor therapy; (4) Eastern Cooperative Oncology Group performance status (ECOG-PS) score of 0–2; (5) Expected survival time of ≥ 6 months with sufficient organ and bone marrow function; (6) Immunotherapy cycles ≥ 4 cycles. Exclusion criteria: (1) Included a history of other malignancies, active autoimmune diseases, prior non-infectious pneumonia or interstitial lung disease, uncontrolled diabetes, or congestive heart failure or others.

### Combined chemoradiotherapy regimen

Patients received concurrent chemotherapy during irradiation, with 56 patients receiving radiotherapy for all lesions involving the esophagus or metastatic lymph nodes (Radiation for all lesions, RAL). Another 23 patients received partial lesion irradiation (Radiation for partial lesions, RPL), including 5 with esophageal lesion irradiation only, 3 with abdominal lymph node irradiation only, 1 with brain metastasis irradiation only, and 14 with irradiation of esophageal lesions accompanied by regional metastatic lymph nodes. All patients received intensity-modulated radiation therapy, with a prescription dose of 50.4–60.0 Gy in 28–30 fractions, 1.8–2.0 Gy per fraction, 5 times per week. Dose constraints for critical organs included bilateral lung average dose < 15 Gy, lung V20 (percentage volume receiving ≥ 20 Gy) < 28%, lung V5 (percentage volume receiving ≥ 5 Gy) < 55%, heart mean dose ≤ 26 Gy, heart V30 (percentage volume receiving ≥ 30 Gy) < 35%, maximum spinal cord dose < 45 Gy, maximum brainstem dose < 54 Gy, and liver V30 (percentage volume receiving ≥ 30 Gy) < 30%. All patients completed irradiation treatment as planned. Chemotherapy regimens were platinum-based, including albumin-bound paclitaxel, paclitaxel, and 5-fluorouracil, with 2–6 cycles and a median of 4 cycles. Immunotherapies included Camrelizumab, Tirelizumab, Toripalimab, and Pembrolizumab, with utilization rates of 31 cases (39.2%), 25 cases (31.6%), 14 cases (17.7%), and 9 cases (11.4%), respectively. The median number of immunotherapy cycles was 6, administered every 3 weeks or until disease progression, unacceptable toxicity, clinical decision, or patient refusal. Patients were categorized into three groups based on the sequence of ICIs and CRT application: ICIs after CRT group (Group 1), CRT contemporaneous ICIs group, and CRT after ICIs group (Group 3).

### Lymphocyte count test and classification

Patients undergo routine blood tests before treatment (baseline), mid-treatment (defined as after 15 sessions of radiotherapy), and post-treatment (defined as 1 month after the completion of radiotherapy). Patients are grouped based on the lymphocyte count. The classification is defined as follows: High (lymphocyte count: > 1*10^9/L), Medium (lymphocyte count: 0.5 ~ 1*10^9/L), Low (lymphocyte count: < 0.5*10^9/L).

### Assessment of efficacy and treatment-related adverse events

Adverse events and laboratory test results were assessed weekly during chemoradiotherapy and every 3 weeks during immunotherapy. Efficacy assessment was based on the Response Evaluation Criteria In Solid Tumors (irRECIST) version 1.1 and classified as complete response (CR), partial response (PR), stable disease (SD), or progressive disease (PD). Overall response rate (ORR) was calculated as the percentage of patients achieving CR and PR among the total. Disease control rate (DOR) was calculated as the percentage of patients achieving CR, PR, and SD among the total. Treatment-related adverse events (trAEs) were graded according to the National Cancer Institute Common Terminology Criteria for Adverse Events (NCI-CTCAE) version 5.0.

### Follow-up and recurrence assessment

Regular follow-up examinations were conducted for enrolled patients, including blood tests, chest and abdominal CT scans, esophagography, and superficial lymph node ultrasound. Esophageal recurrence was confirmed by pathological examination through endoscopic biopsy, and superficial lymph node recurrence was confirmed by fine-needle aspiration biopsy when necessary. Follow-up assessments were conducted monthly in the first year, every 3 months in the second year, and every 6 months thereafter. The last follow-up date was December 31, 2022, with a follow-up rate of 100%. Recurrence was categorized as local or distant progression based on histopathology, cytology, or clear imaging evidence, with local recurrence defined as esophageal or regional lymph node recurrence and distant recurrence as non-regional lymph node recurrence or organ metastasis.

### Statistical analysis

Statistical analysis was performed using SPSS 26.0 software. Differences in categorical variables were assessed using the chi-square or Fisher’s exact test, while the Mann–Whitney U test was used for continuous variables. Kaplan–Meier methods were employed to estimate overall survival (OS) and progression-free survival (PFS), with significance determined by log-rank tests for survival differences between groups. Multivariate analysis was conducted for variables with *P* ≤ 0.05 in univariate analysis, using the Cox proportional hazards regression model to estimate hazard ratios (HR) and 95% confidence intervals (CI). Median follow-up was calculated using the Kaplan–Meier method. All tests were two-tailed, with *P* < 0.05 considered statistically significant. The primary endpoints of this study were OS and PFS, defined as the time from the start of treatment to the date of death or the last follow-up.

## Results

### General characteristics

From January 2018 to March 2021, a total of 79 eligible patients were included in this retrospective real-world study. 73.4% were male, with a median age of 66 years. Among the patients, 14 had a baseline ECOG-PS score of 0, and 68.4% had scores of 0–1. 8.1% (7 cases) were cervical ESCC, while the remaining 91.1% had thoracic ESCC. Histological differentiation revealed 20 cases of poorly differentiated tumors, with 74.7% classified as moderately or well-differentiated. There were 14, 36, and 19 patients in stages II, III, and IV, respectively. Among the 19 stage IV patients, 7 had distant organ metastases, while the remaining 12 had distant lymph node metastases.

### Prognostic analysis

The median follow-up time for the entire cohort was 29.4 months. The 1-year, 2-year, and 3-year OS rates were 85.7%, 62.3%, and 56.2%, respectively, and the 1-year, 2-year, and 3-year PFS rates were 62.7%, 30.2%, and 26.5%, respectively. The median OS was not reached, while the median PFS was 14.6 months (95% CI 10.7–18.5). Short term efficacy showed 4 cases of CR, 61 cases of PR, 11 cases of SD, and 3 cases of PD. The ORR was 82.3% (65/79), and the DOR was 96.2% (76/79).

### Univariate analysis of factors affecting prognosis

As shown in Table 1, factors significantly affecting OS included clinical stage, number of immunotherapy cycles, sequence of immunotherapy and CRT, and short term efficacy (χ^2^ = 6.590, 8.408, 10.345, 7.762; *P* = 0.010, 0.004, 0.001, 0.005). Factors significantly affecting PFS included clinical stage, sequence of immunotherapy and CRT, and short term efficacy (χ^2^ = 9.360, 7.045, 4.449; *P* = 0.002, 0.008, 0.035). See Table 1 for details.

**Table 1:** Univariate analysis for factor affecting prognosis.

	N	OS rate (%)			X^2^	*P*	PFS rate (%)			X^2^	*P*
		1 year	2 year	3 year			1 year	2 year	3 year		
Sex					3.067	0.080				0.031	0.860
Male	58	84.1	57.1	48.8			60.1	29.2	27.1		
Female	21	90.2	78.2	78.2			70.7	32.7	21.8		
Age (Year)					0.032	0.857				0.179	0.672
≤ 66	41	82.9	62.6	54.8			63.4	32.3	29.6		
> 66	38	89.3	61.3	57.7			62.2	27.6	22.1		
Tumor location					3.036	0.219				0.732	0.693
Cervical/upper thoracic	24	82.9	68.5	68.5			70.3	37.5	37.5		
Middle thoracic	37	86.3	67.0	55.5			67.2	26.2	18.3		
Lower thoracic	18	88.1	44.1	37.8			44.4	27.8	27.8		
ECOG score					3.492	0.062				1.997	0.158
0–1	54	86.4	68.4	68.4			69.7	35.6	29.5		
2	25	84.0	52.0	38.5			48.0	20.0	20.0		
Histologic grade					0.685	0.408				0.251	0.616
Poorly differentiated	20	95.0	66.6	66.6			50.0	33.3	26.7		
Moderate/Highly Differentiated	59	82.5	60.7	52.9			67.3	29.5	26.5		
Clinical stage					6.590	0.010				9.360	0.002
II + III	50	89.8	70.9	68.4			79.9	37.6	32.1		
IV	29	78.9	47.4	35.9			33.0	17.6	17.6		
Number of immune cycles (cycles)					8.408	0.004				2.765	0.096
≤ 6	44	74.4	45.6	45.6			54.0	26.9	16.0		
≥ 7	35	100	83.3	70.5			73.7	33.9	33.9		
Chemotherapy cycles					0.001	0.974				0.025	0.875
≤ 3	39	78.9	61.1	61.1			66.3	32.4	27.8		
≥ 4	40	92.4	63.3	51.6			59.5	28.0	24.9		
Radiation coverage					7.087	0.008				0.262	0.609
All lesion	56	87.2	70.2	65.8			67.5	30.9	25.5		
Partial lesion	23	82.1	43.5	33.8			51.0	27.8	27.8		
ICIs sequence					10.345	0.001				7.045	0.008
Sequential	50	98.0	76.2	66.3			69.4	42.4	36.9		
Concurrent	29	65.2	39.8	39.8			51.3	11.0	11.0		
Short term efficacy					7.762	0.005				4.449	0.035
CR + PR	65	87.4	69.7	62.3			65.8	33.7	29.5		
SD + PD	14	77.9	26.3	26.3			49.0	12.2	12.2		

Complete response (CR), partial response (PR), immune checkpoint inhibitors (ICIs)

### Analysis of the impact of ALC changes on patient prognosis

The baseline ALC, mid-treatment ALC, and post-treatment ALC for the entire cohort were in the ranges of 0.23–2.96 × 10^9/L [(1.425 ± 0.602) × 10^9/L], 0.17–2.25 × 10^9/L [(1.039 ± 0.521) × 10^9/L], and 0.06–2.83 × 10^9/L [(0.924 ± 0.532) × 10^9/L], respectively. Significant differences were observed between baseline ALC and mid-treatment ALC, as well as between baseline ALC and post-treatment ALC (t = 4.006, 4.896; *P* < 0.001), while no significant difference was noted between mid-treatment ALC and post-treatment ALC (t = 1.188, *P* = 0.237).

**Fig. 1:**
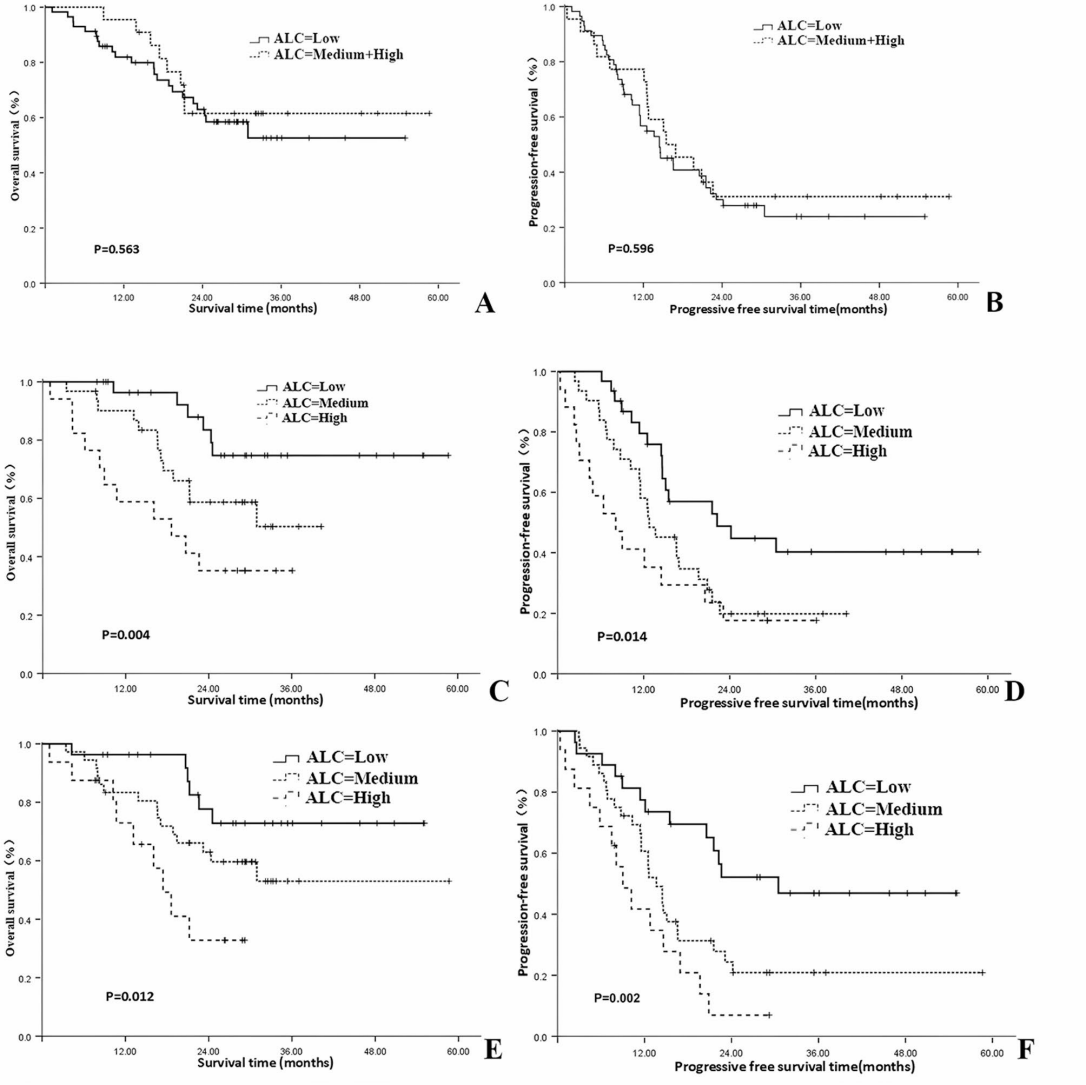
Analysis of the Impact of absolute lymphocyte count (ALC) Levels at different time periods on prognosis; **A** Baseline ALC for overall survival; **B** Baseline ALC for progression free survival; **C** Mid-treatment ALC for overall survival; **D** Mid-treatment ALC for progression free survival; **E** Post-treatment ALC for overall survival; **F** Post-treatment ALC for progression free survival.

Further analysis revealed that ALC levels at different time points had varying impacts on patient prognosis. Baseline ALC had no significant influence on both OS and PFS (χ^2^ = 0.335, 0.281;* P* = 0.563, 0.596). In contrast, lower mid-treatment ALC and post-treatment ALC were adverse factors for OS (χ^2^ = 10.939, 8.778; *P* = 0.004, 0.012) and PFS (χ^2^ = 8.472, 12.949; *P* = 0.014, 0.002). See Table 2 and Fig. 1 for details.

**Table 2:** Analysis of the impact of ALC levels at different time periods on patient prognosis.

Index	N	OS rate (%)			X^2^	*P*	PFS rate (%)			X^2^	*P*
		1 year	2 year	3 year			1 year	2 year	3 year		
Baseline ALC					0.335	0.563				0.281	0.596
Low	57	81.9	62.9	52.6			56.8	30.1	23.9		
Medium + High	22	95.5	61.5	61.5			77.3	31.2	31.2		
Mid-treatment ALC					10.939	0.004				8.472	0.014
Low	31	96.3	83.5	74.7			79.5	48.8	40.3		
Medium	31	90.1	58.7	50.3			58.1	19.9	19.9		
High	17	58.8	35.3	35.3			41.2	17.6	17.6		
Post-treatment ALC					8.778	0.012				12.949	0.002
Low	27	96.3	77.7	72.8			77.4	52.1	46.9		
Medium	36	83.3	62.9	53.0			60.7	24.3	20.9		
High	16	72.9	32.8	32.7			41.7	6.9	6.9		

*ACL* absolute lymphocyte count.

### The impact of different irradiation coverage on patient prognosis

**Fig. 2:**
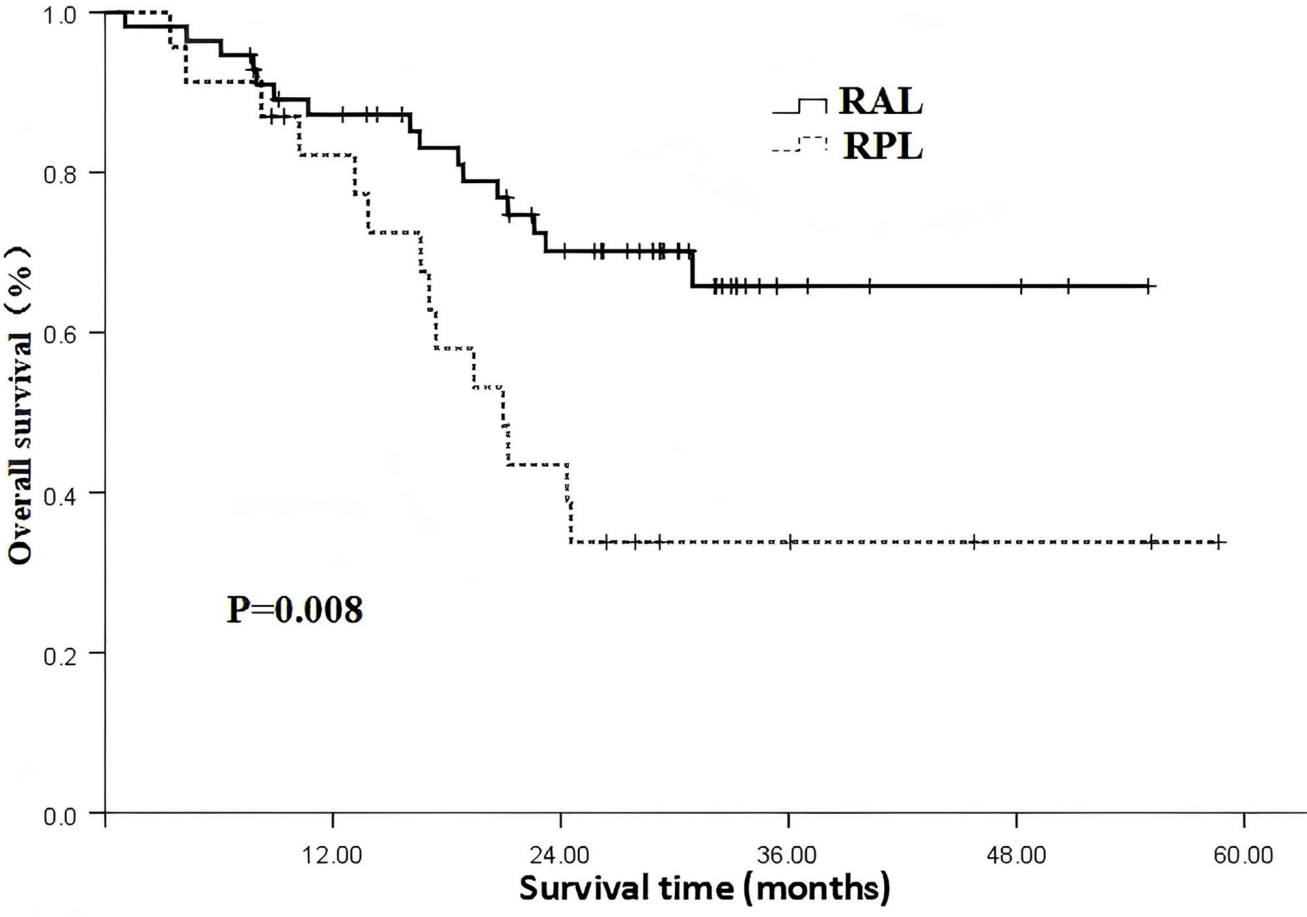
The impact of radiation coverage on overall survival (RAL: Radiation for all leisions; RPL: Radiation for partial leisions).

**Fig. 3:**
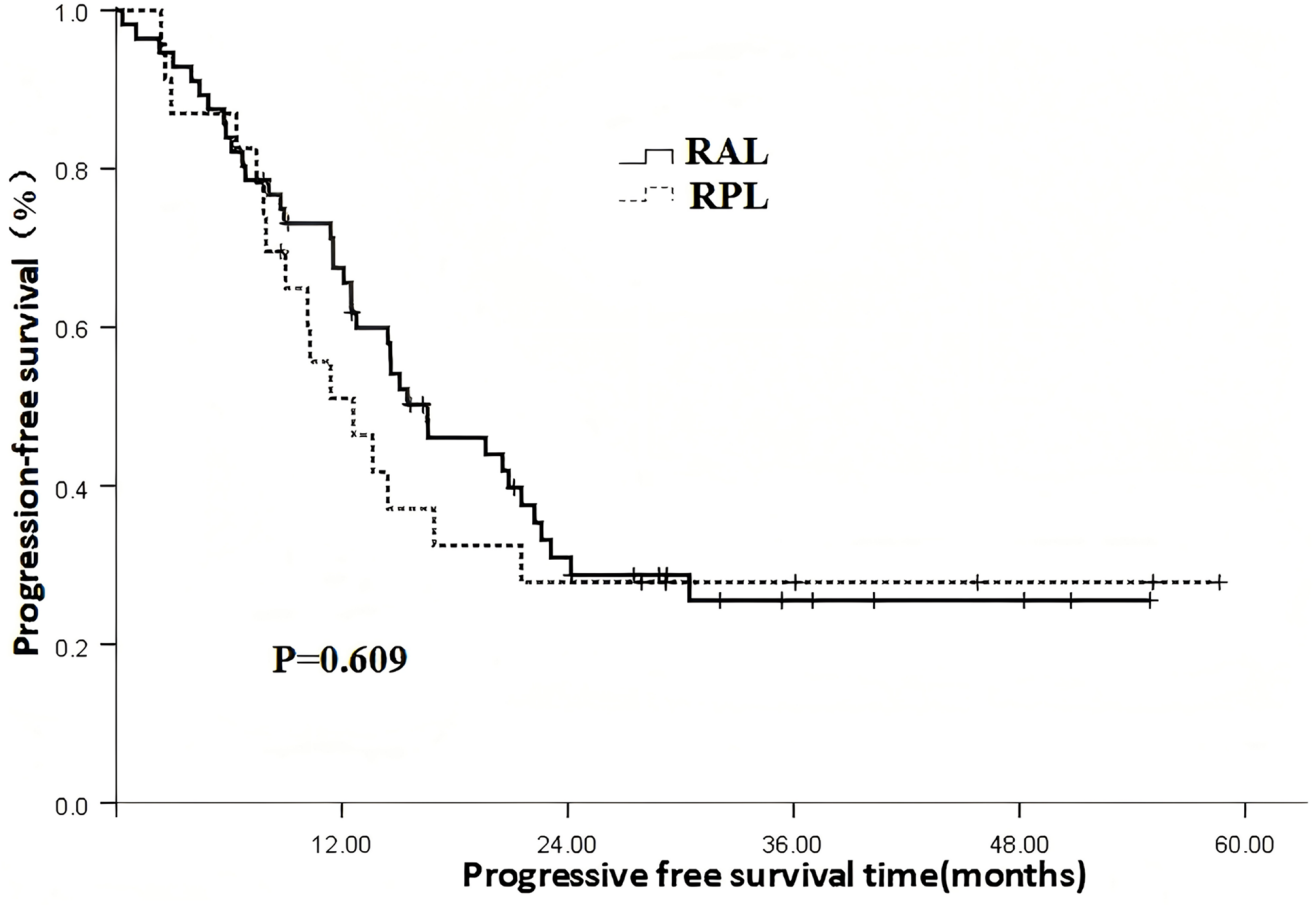
The impact of radiation coverage on progression free survival (RAL: Radiation for all leisions; RPL: Radiation for partial leisions)

The results show that the irradiation coverage is a significant factor affecting patient OS, as indicated in Table 3 (χ^2^ = 7.087, *P*=0.008). However, it does not have a significant impact on PFS, as shown in Table 3 and Figs. 2, 3 (χ^2^ = 0.262, *P* = 0.609). The results showed that patients receiving all leisions radiotherapy had significantly longer OS and PFS than those with partial leisions radiotherapy.

**Table 3:** Analysis of the impact of different irradiation coverage on patient prognosis.

Index	N	OS rate (%)			X^2^	*P*	PFS rate (%)			X^2^	*P*
		1 year	2 year	3 year			1 year	2 year	3 year		
Radiation Coverage					7.087	0.008				0.262	0.609
All lesion	56	87.2	70.2	65.8			67.5	30.9	25.5		
Partial lesion	23	82.1	43.5	33.8			51.0	27.8	27.8

### Analysis of the impact of immunotherapy and CRT sequence on patient prognosis

There were 20 cases in the "ICIs after CRT" group (Group 1), with time interval ranged from 0.5 to 5.93 months, with a median of 2.0 months. 29 cases in the "CRT contemporaneous ICIs" group (Group 2), and 30 cases in the “CRT after ICIs group ” (Group 3), with time interval ranged from 0.7 to 6.0 months, with a median of 1.95 months. The results presented in Table 4 indicate that, overall, the different sequences of ICIs combined with CRT had a significant impact on both patient OS and PFS (χ^2^ = 10.502, 7.045; *P* = 0.005, 0.030). Further pairwise comparisons revealed the following:

**Table 4:** Analysis of the impact of immunotherapy sequence on patient prognosis.

Index	N	OS rate (%)			X^2^	*P*	PFS rate (%)			X^2^	*P*
		1 year	2 year	3 year			1 year	2 year	3 year		
Immunotherapy sequence					10.501	0.005				7.045	0.030
Group 1: ICIs after CRT	30	100.0	79.2	69.1			73.3	39.7	36.1		
Group 2: CRT contemporaneous ICIs	29	65.2	39.8	39.8			51.3	11.0	11.0		
Group 3:CRT after ICIs	20	95.0	70.4	60.3			62.6	50.0	37.9		

*OS* Overall survival, *PFS* progression free survival, *CRT* chemoradiotherapy, *ICIs* immune checkpoint inhibitors.

Group 1 vs. Group 2: There was a significant difference in OS (χ^2^ = 8.928, *P* = 0.003) but no significant difference in PFS (χ^2^ = 5.537, *P* = 0.019).

Group 1 vs. Group 3: There was no significant difference in OS (χ^2^ = 0.558, *P* = 0.445) or PFS (χ^2^ = 0.001, *P* = 0.975).

Group 2 vs. Group 3: There was a significant difference in PFS (χ^2^ = 4.183, *P* = 0.041).

**Fig. 4:**
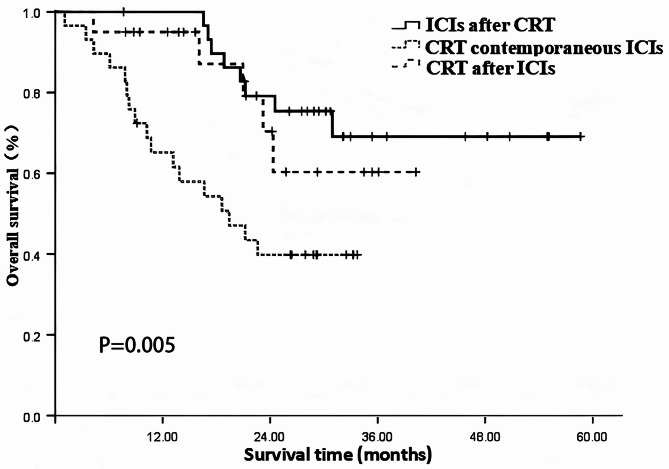
The impact of Immune checkpoint inhibitors (ICIs) and CRT (Chemoradiotherapy) Sequence on overall survival.

**Fig. 5:**
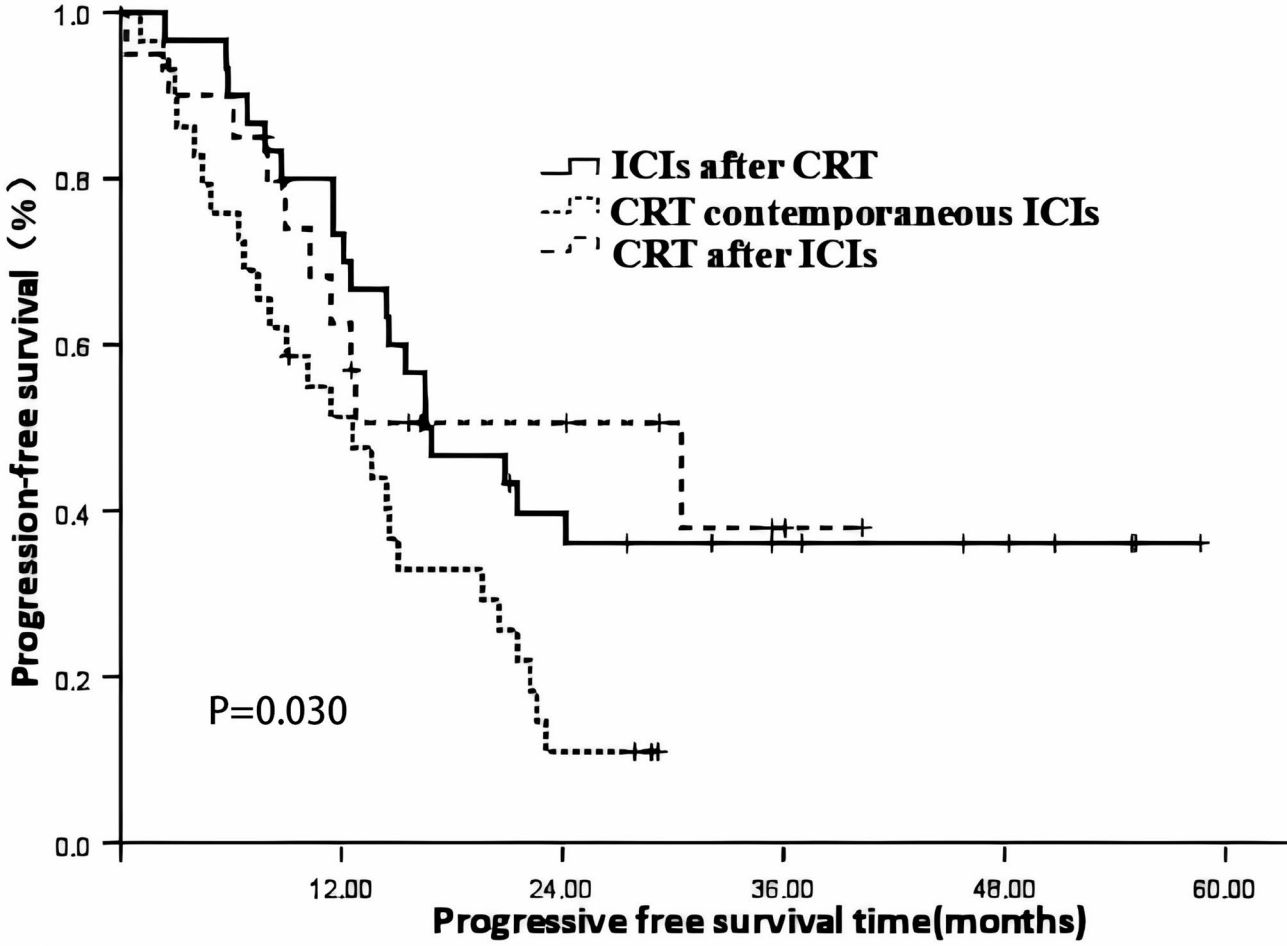
The impact of Immune checkpoint inhibitors (ICIs) and CRT (Chemoradiotherapy) Sequence on progression free survival.

The results indicated a better prognosis of sequential chemoradiotherapy and ICIs (group 1 and group 3) than concurrent chemoradiotherapy and ICIs (group 2). See Table 4 and Figs. 4, 5 for details.

### Multifactorial analysis of prognostic factors

As shown in Table 5, independent prognostic factors for patient OS included clinical stage, number of immunotherapy cycles, sequence of immunotherapy and CRT, radiation coverage, mid-treatment ALC level, and short term efficacy (HR = 2.254, 0.374, 2.653, 2.957, 2.309, 2.789; *P* = 0.030, 0.019, 0.009, 0.004, 0.001, 0.014). Independent prognostic factors for PFS included clinical stage, sequence of immunotherapy and CRT, and post-treatment ALC (HR = 2.135, 2.048, 1.911; *P* = 0.007, 0.010, 0.001). Please refer to Table 5 for more details.

**Table 5:** Multifactorial analysis of prognostic factors.

Index	Factor	Regression coefficient	Standard error	χ^2^	*P* value	HR	95%CI	
							Lower limit	Upper limit
OS								
Clinical stage	II + III/IV	0.813	0.374	4.711	0.030	2.254	1.082	4.695
Number of immunotherapy	≤ 6/ ≥ 7 cycles	− 0.984	0.420	5.500	0.019	0.374	0.164	0.851
Immunotherapy Sequence	Sequential/Concenrent	0.976	0.374	6.798	0.009	2.653	1.274	5.525
Radiation coverage	All/Partial	1.084	0.376	8.298	0.004	2.957	1.414	6.184
Mid-treatment ALC	Low/Medium/High	0.837	0.256	10.669	0.001	2.309	1.398	3.816
Short term efficacy	CR + PR/SD + PD	1.026	0.420	5.977	0.014	2.789	1.226	6.346
PFS								
Clinical stage	II + III/IV	0.759	0.282	7.235	0.007	2.135	1.229	3.711
Immunotherapy sequence	Sequential/Concenrent	0.717	0.277	6.724	0.010	2.048	1.191	3.522
Post-treatment ALC	Low/Medium/High	0.648	0.197	10.822	0.001	1.911	1.299	2.811

*HR* Hazard ratio, *OS* Overall survival, *PFS* Progression free survival, *ALC* Absolute lymphocyte count

### Analysis of treatment failure patterns and retreatment method

As of the last follow-up date, a total of 37 patients in the entire group experienced treatment failure. Among them, 9 cases had primary lesion recurrence, 18 cases developed new lesions, and 10 cases had primary lesion recurrence accompanied by the appearance of new lesions. Out of the 37 cases, 33 patients received second-line treatment, with only 4 patients undergoing conservative management. The 1-year and 2-year OS rates for these patients were 56.7% and 24.3%, with a median OS of 14.17 months (95% CI 7.303 ~ 21.037). See Table 6 for details.

**Table 6:** Patient treatment failure patterns and retreatment modalites.

Failure Patterns	Failure sites	Retreatment modalites	Survival status
Primary leision	Primary esophageal cancer recurrence: 5 cases	CT + ICIs:3 cases	5 cases survival
Recurrence (N = 9)	Mediastinal lymph node recurrence: 1 case	RT: 2 cases	4 cases death
	Esophageal and mediastinal lymph node recurrence: 1 case	Apatinib + ICIs: 1 case	
	Esophageal with mediastinal and abdominal lymph nodes recurred: 1 case	ICI only: 1 case	
		Conservative treatment: 2 cases	
Occurence of new leision (N = 18)	Bone metastasis: 6 cases	CRT + ICIs:3 cases	7 cases survival
	Liver metastasis: 2 cases	CT + ICIs:7 cases	11 cases death
	Brain metastasis: 2 cases	CRT + ICIs: 2 cases	
	Lung metastasis: 1 case	CT + ICIs + targeted drugs:2 cases	
	Mediastinal lymph node recurrence: 3 cases	RT: 3 cases	
	Supraclavicular Lymph node metastasis: 2 cases	ICI: 2 cases	
	Abdominal lymph nodes recurred: 1 case	CT + ICIs + radiofrequency:1 case	
	Lymph node, liver, bone metastasis: 1 case	Conservative treatment: 1 case	
Primary leision recurrence + Occurence of new leision (N = 10)	Esophageal Recurrence with Lung, Vertebral, Supraclavicular, and Abdominal Lymph Node Involvement (1 Case)	CRT + ICIs: 2 cases	5 cases survival
	Mediastinal Lymph Node Recurrence with Brain Metastasis (1 Case)	CT + ICIs: 2 cases	5 cases death
	Mediastinal Lymph Node Recurrence with Supraclavicular and Abdominal Lymph Node Involvement (1 Case):	ICIs: 2 cases	
	Mediastinal Lymph Node Recurrence with Abdominal Lymph Node Involvement (2 Cases)	RT + ICIs: 1 case	
	Neck and Abdominal Lymph Node Involvement with Liver Metastasis (1 Case)	RT: 1 case	
	Esophagus with abdominal lymph node recurrence: 1 case	S-1: 1 case	
	Abdominal lymphatic and adrenal metastasis: 1 case		
	Primary recurrence with multiple: 2 cases	Conservative treatment: 1 case	

*ICIs* immune checkpoint inhibitors, *CRT* chemoradiotherapy, *RT* radiotherapy, *CT* chemotherapy.

### Analysis of trAEs

 During the course of treatment, all patients experienced trAEs of any grade. Among them, 29 patients (36.7%) experienced trAEs of grade ≥ 2, and no grade 5 adverse events were observed. The most common grade ≥ 3 trAEs were bone marrow suppression, including isolated bone marrow suppression in 7 cases, 1 case accompanied by esophagitis and pneumonia, and 1 case accompanied by pneumonia, esophagitis, gastrointestinal bleeding, esophageal fistula, peripheral neuropathy, and abnormal liver function, each. Immune myocarditis occurred in 3 cases, including 1 case of isolated immune myocarditis, 1 case with liver and kidney dysfunction, and 1 case with pneumonia. Additionally, 12 cases (15.2%) experienced grade ≥ 2 trAEs, including isolated pneumonia in 3 cases, isolated esophageal fistula in 3 cases, isolated esophagitis in 2 cases, cardiac dysfunction in 2 cases, gastrointestinal bleeding in 1 case, and hyperthyroidism in 1 case. Among the 79 patients, a total of 4 cases (5.1%) developed esophageal fistulas. Further analysis revealed that 2 of these 4 patients were in T4 stage, while the other 2 had baseline malnutrition with albumin levels < 30 g/L.

## Discussion

Esophageal cancer is a significant threat to human health^12^. In recent years, with improvements in diagnostic and treatment, there has been significant progress in the overall survival of esophageal cancer. However, the outcomes are still not ideal. For non-surgical esophageal cancer patients, the NCCN guidelines recommend definitive chemoradiotherapy as the primary treatment for those without metastasis. Based on pooled data from multiple clinical studies, the 3-year survival rate for patients, primarily with stage II–III disease, receiving conventional definitive chemoradiotherapy is approximately 22.4–45.5%^13–17^. For patients with distant metastasis, phase III clinical trials such as KEYNOTE-590 and CheckMate 648 have established chemotherapy combined with immunotherapy as the guideline-recommended treatment, with an estimated 3-year survival rate of 15–25%^18,19^. However, whether the triplet regimen of chemoradiotherapy plus immunotherapy provides better survival outcomes remains uncertain. To enhance treatment effectiveness, clinicians have explored combinations of immunotherapy with other treatments, and increasing evidence suggests that immunotherapy combined with other treatments can significantly improve the prognosis of esophageal cancer patients. ICIs in combination with chemotherapy have shown superiority in both first-line and second-line treatment of esophageal cancer^4–9^. In our study, the 3-year survival rate was 56.2%, which is higher than most reported data on conventional chemoradiotherapy. Considering that our study cohort included a relatively high proportion (36.7%) of clinical stage IV patients, this survival outcome is encouraging. It suggests that the combination of immunotherapy with chemoradiotherapy is a promising treatment approach that warrants further exploration and analysis across different subgroup of esophageal cancer.

Clinical research suggests that aggressive local intervention through radiotherapy can improve the obstruction symptoms in esophageal cancer patients, thereby improving their nutritional status, enhancing their quality of life, and stimulating systemic anti-tumor immune responses, leading to better outcomes^20,21^. The PACIFIC regimen has achieved good clinical efficacy in non-small cell lung cancer. Whether this outcome can be replicated in patients with esophageal cancer requires large-scale, prospective clinical research to answer. Oligometastasis is relatively common in patients with ESCC, which suggests the importance of combining local and systemic treatments in the management of ESCC^22^. In this retrospective analysis of 79 ESCC patients who received ICIs in combination with concurrent chemoradiotherapy , the results showed that the treatment outcome was good. The median OS was not reached, and the median PFS was 14.6 months. The ORR was 82.3%, and the DOR was 96.2%.

Currently, there are limited prospective studies on ICIs in combination with CRT for the treatment of ESCC, and these studies typically report survival outcomes at 1 to 2 years. Zhu et al. conducted a single-arm phase II clinical trial of dCRT in combination with Toripalimab for advanced ESCC (EC-CRT-001), and among 42 patients, 26 achieved complete responses, with 1-year OS and PFS rates of 78.4% and 54.5%, respectively. Zhang et al. conducted a phase Ib study of ICIs in combination with radiation therapy, reporting 1- and 2-year OS rates of 85.0% and 69.6%, and PFS rates of 80.0% and 65.0%, respectively. The median OS and PFS times were 16.7 months and 11.7 months, respectively, with an ORR of 74%. Another phase II clinical trial reported by Park et al. used CRT in combination with durvalumab and tremelimumab to treat 40 patients, resulting in 2-year OS and PFS rates of 75% and 57.5%, respectively, with greater benefits observed in patients with high PD-L1 expression. The relatively higher PFS rates observed in the studies mentioned compared to the current study could potentially be attributed to the inclusion of stage IV patients in the present study^23–25^.

Combining CRT with ICIs in the treatment of ESCC is still associated with several uncertainties, and the sequence of CRT and ICIs is one such aspect. Most phase III studies in clinical practice have adopted concurrent ICIs and CRT^26,27^. In contrast, the TENERGY study design^28^ involved CRT followed by sequential application of atezolizumab for consolidative immunotherapy. The results of this study show that the timing sequence of ICIs and CRT has a significant impact on survival. Pairwise comparisons show that sequential treatment with CRT and ICIs is more effective than concurrent treatment. The optimal combination strategy for CRT and ICIs in esophageal cancer treatment necessitates further clinical trials for validation. Although the optimization of radiotherapy techniques, anti-tumor drugs, dosages, and treatment duration remains unclear, continuous data collection and analysis from clinical trials (such as NCT 03278626, NCT 02520453, NCT 03377400, NCT032786626, and NCT 02844075) can offer insights into the efficacy of combined CRT and ICIs^29^.

Lymphocytes are a critical component of the human immune system, playing a pivotal role in preventing the occurrence and development of tumors. A decrease in the lymphocyte count in the human body often indicates an immune-suppressed state, leading to poor responses to anti-tumor treatments^30^. Especially in the era of immunotherapy, lymphocyte counts are garnering increased attention in clinical practice. However, the impact of combined CRT and ICIs on treatment-related lymphocyte reduction remains unclear. Furthermore, the potential risk factors associated with lymphocyte reduction in ESCC patients undergoing combined ICIs and CRT have not been thoroughly studied. Prior research consistently indicates that patients with lower ALC exhibit significantly reduced responses to immunotherapy and significantly poorer prognoses^31,32^.Cheng et al.^33^ conducted an analysis of 602 ESCC patients receiving definitive CRT, of which 166 received ICIs in combination with CRT. Their study aimed to compare ALC changes and survival rates between patients who received ICIs and those who did not during CRT. Their research indicated that patients with lower ALC levels during CRT often had worse outcomes with ICIs treatment. ALC reduction might lead to a decrease in the efficacy of ICIs and reduced ICIs therapy efficacy, ultimately resulting in an inferior prognosis. Our study also shows that as treatment progresses, there is a significant reduction in ALC levels, and lower ALC levels are significantly associated with poorer patient outcomes. We also noted that the composition of the patient population did not significantly differ in terms of ALC changes during mid-treatment and post-treatment phases. To some extent, this suggests that patients receiving ICIs treatment do not experience a pronounced decline in lymphocyte counts during treatment, implying a protective effect of ICIs on lymphocytes. This could be another manifestation of the synergy between radiotherapy and ICIs treatment, which aligns with the findings of Zhu et al. ^23^. Therefore, we believe that in the early stages of immunotherapy, lymphocyte counts may temporarily decrease, but if this decline continues over time, it should be a cause for significant concern. Incorporating ICIs treatment into dCRT can help mitigate the decline in ALC.

Currently, the coverage of radiation therapy in patients receiving ICIs in combination with CRT is a noteworthy topic for exploration. Radiation therapy is one of the factors contributing to a reduction in ALC. A larger radiation coverage generally implies a higher likelihood of impacting lymphocyte-generating sites and organs with a high blood flow, potentially increasing the occurrence of lymphocyte reduction^33,34^. In clinical practice, elective nodal irradiation was widely accepted for locally advanced ESCC. However, for metastatic ESCC, there is limited research regarding whether selective irradiation of specific lesions or irradiation of all lesions is more appropriate. In the cases of lung cancer, it is often suggested that irradiation of primary lesions and oligometastatic lesions simultaneously can improve patient outcomes^35,36^. In our study, 29 patients were diagnosed with stage IV ESCC, with 23 of them receiving partial lesion radiation therapy. The results indicate that patients receiving partial lesion irradiation experienced worse outcomes, which may be attributed to these patients being in stage IV. Unfortunately, due to the relatively small number of cases in our study, we were unable to perform a comparative analysis of different irradiation coverage for stage IV patients. The addition of localized treatment to systemic therapy can effectively improve patient prognosis, and this enhancement is likely associated with several mechanisms. Firstly, localized treatment can be directed at the residual tumor areas after systemic therapy, potentially reducing the occurrence of distant micrometastatic lesions during treatment course^37^. Secondly, localized treatment can enhance the sensitivity of systemic chemotherapy. Lastly, by reducing the overall tumor cell burden, localized treatment may lead to fewer distant micrometastatic lesions^38^.

The occurrence of trAEs is a major concern for clinicians. In our study, aside from some hematological complications, the combination of ICIs and CRT did not significantly increase the incidence or severity of trAEs in patients. Grade 2 or higher trAEs included pneumonia, esophagitis, which is in line with the findings of prior research on late-stage ESCC patients treated with ICIs combined therapy^5,6,8^. Notably, our cohort included 4 cases of esophageal fistula, with an incidence rate of 5.1%. Similarly, Ma et al.^39^ reported an incidence rate of 3.3% for esophageal fistula, Zhu et al.^19^ observed a 14% incidence rate for grade 2 or 3 esophageal fistula (6/42), and Zhang et al.^20^ reported a 10.5% incidence rate (2/19). ESCC patients who previously underwent radical radiotherapy and combined chemotherapy had esophageal fistula incidence rates ranging from 5.6 to 33%^40,41^. Based on those existing data, we consider the incidence of esophageal fistula in ESCC patients undergoing ICIs combined with chemoradiotherapy to be acceptable. Clinically, the primary risk factors associated with esophageal fistula include patient age, ECOG performance status, clinical T-stage, poor treatment response, combined therapy, ulcerative esophageal cancer, nutritional status, and recurrence with subsequent radiation therapy.We recommend vigilance in monitoring the occurrence of esophageal fistula in esophageal cancer patients receiving ICIs combined with CRT, especially in those with these aforementioned risk factors. Furthermore, even in ESCC patients who have undergone ICIs combined with CRT, the primary failure pattern was local recurrence or distant metastasis. In our study, the incidence rates of local recurrence, distant metastasis, both local recurrence and distant metastasis rate were 11.4%, 22.8% and 12.7% respectively, these patients generally exhibit favorable outcomes after second-line treatment, with a median overall survival time of 14.17 months (95% CI 7.303–21.037). Currently, there is limited research on the failure patterns of ESCC patients treated with ICIs combined with CRT. Based on the results of our study, it is advisable to actively pursue second-line treatment for patients who experience treatment failure after first-line ICIs + CRT therapy.

Due to the retrospective nature of this study conducted in a single center, there are inherent risks of selection bias and misclassification bias. Additionally, data regarding the treatment status and time of death for patients were obtained during follow-up, which may introduce recall bias. Furthermore, there was variation in chemotherapy/immunotherapy regimens and radiation dosages within treatment modality. Moreover, due to the limited number of patients in this study who underwent pretreatment PD-L1 expression testing (only 13 cases), we were unable to evaluate whether pretreatment PD-L1 expression levels influence patient prognosis. Therefore, the results of this study require confirmation through larger sample sizes and longer-term follow-up in multicenter, prospective research.

In conclusion, our study findings suggest that the combination of ICIs with CRT can improve the efficacy and prognosis of patients with locally advanced and recurrent/metastatic ESCC while maintaining manageable side effects. The radiation for all leisions, sequential immunotherapy combined with chemoradiotherapy, and higher levels of lymph node cell counts are associated with a better prognosis. This study provides a necessary basis for further in-depth research and suggests that this treatment approach warrants further investigation in large-scale, randomized controlled clinical trials.

## Data Availability

Research data are stored in an institutional repository and will be shared upon request by the corresponding author.

## Abbreviations

ESCC: Esophageal squamous cell carcinoma
CRT: Chemoradiotherapy
ICIs: Immune checkpoint inhibitors
ALC: Absolute lymphocyte count
ECOG-PS: Eastern Cooperative Oncology Group performance status
OS: Overall survival
PFS: Progression-free survival
ORR: Objective response rate
DOR: Disease control rate
trAEs: Treatment-related advers
RAL: Radiation for all leisions;
RPL: Radiation for partial leisions
